# Analysis of trends and prospects regarding stents for human blood vessels

**DOI:** 10.1186/s40824-018-0114-1

**Published:** 2018-03-13

**Authors:** Jeong Hee Lee, Eung Do Kim, Eun Jung Jun, Hyoung Sun Yoo, Joon Woo Lee

**Affiliations:** 1 Graduate School of Health Science Business Convergence, College of Medicine, Chungbuk National University, Chungdae-ro 1, Seowon-Gu, Cheongju, Chungbuk 28644 Korea; 2 Aurios Medical, M-2503, 32 569 Songdogwahak-ro, Yeonsu-gu, Incheon, 21984 Korea; 3 Korea Institute of Science and Technology Information, 66 Heogi-ro, 570 Dongdeamoon-gu, Seoul, 02456 Korea

**Keywords:** Vascular stent, Coronary artery stent, Drug-eluting stents (DES), Bioresorbable scaffolds (BRS), Vascular occlusion, Restenosis, Implantable medical devices, Market analysis

## Abstract

**Background:**

The purpose of this paper is to provide technology trends and information regarding market and prospects in stents used for human blood vessels in Korea and the world.

A stent is a medical device in the form of a cylindrical metal net used to normalize flow when blood or other bodily fluids such as biliary fluids are obstructed in blood vessels, gastrointestinal tracts, etc. by inserting the stent into a narrowed or clogged area. Stents are classified into vascular and non-vascular stents. The coronary artery stent is avascular stent that is used for coronary atherosclerosis.

The demand is increasing for stents to treat diseases such as those affecting the heart and blood vessels of elderly and middle-aged patients. Due to the current shift in the demographic structure caused by an aging society, the prospect for stents seems to be very bright.

The use of a stent designed to prevent acute vascular occlusion and restenosis, which is a side effect of conventional balloon angioplasty, has rapidly become popular because it can prevent acute complications and improve clinical outcomes. Since the initial release of this stent, there have been significant developments in its design, the most notable of which has been the introduction of drug-eluting stents (DES). Bioresorbable scaffolds (BRS) have the potential to introduce a paradigm shift in interventional cardiology, a true anatomical and functional “vascular restoration” instead of an artificial stiff tube encased by a persistent metallic foreign body.

**Methods:**

Data for this research were gathered from primary and secondary sources as well as the databases of the Korea Institute of Science Technology Information (KISTI) located in Seoul, Korea like KISTI Market Report. The sources used for primary research included the databases available from the Korea Institute of Science Technology Information, past industry research services/studies, economic and demographic data, and trade and industry journals. Secondary research was used to supplement and complement the primary research. Interviews were conducted with physicians and surgeons from the key hospitals and senior sale/marketing managers from stent product suppliers in South Korea.

**Results:**

The global stent market is estimated at US $ 7.98 billion in 2016 and is expected to grow at a Compound Annual Growth Rate (CAGR) of 3.8% over the next 5 years. As of 2016, the global market for vascular stents is estimated at $ 7.22 billion, with coronary artery stents accounting for 67.3% of the vascular stent market. Among the coronary artery stents, BRS is notably expected to grow at an annual average rate of 8.8% by 2020, but the global adoption rate of BRS remains low at present. In the Korean market, stents for blood vessels account for most of the market, and the market size of stents for blood vessels in Korea was estimated to be 145 billion won as of 2016.

**Conclusions:**

In comparison to the sales growth rate of other medical devices, the future stent technology market is judged to be higher in growth potential.

## Background

Our society’s concerns regarding health is intensifying due to recent global trend toward aging demographics, increase of adult diseases, increase of diseases due to environmental pollution, and the emergence of new viruses. In recent years, there has been a strong emphasis on the importance of prevention and treatment through diagnosis. As the paradigm shifts to wellness, it is expected that the demand for advanced fusion diagnosis and medical devices for treatment will increase [1].

As the population ages, the area of heart disease is one of the therapeutic areas where prevention and treatment are important. Globally, heart disease has been a leading cause of deaths of people aged 60 or older and the number of people with heart disease is increasing. Therefore, the demand for medical devices for treating heart disease is expected to increase. As of 2012, WHO estimates that 37% of deaths from non-communicable diseases are attributable to heart disease and that cardiac mortality rates will increase from 17.5 million in 2012 to 22.2 million in 2030. According to the World Health Organization (WHO), it was estimated that there were about 347 million diabetics in 2014 and the risk of heart disease resulting in death reaches 42% as the blood glucose levels rise [2].

As individuals age, the blood vessels that supply oxygen and nutrients to vital organs such as the brain or heart become clogged up. The most common heart disease is myocardial infarction, which is a clogging of the heart arteries, and angina, which narrows the heart arteries. All of them are emergency medical illnesses associated with sudden death. Symptoms should be treated within 6 to 12 h after the onset of symptoms. In this case, the most commonly used procedure is coronary intervention on arteries which lead to the legs. It is a procedure to insert a cardiovascular stenosis into a blocked part using a metal wire net stent or a balloon [2]. The demand is increasing for stents to treat diseases such as those affecting the heart and blood vessels of elderly and middle-aged people. Due to current shift in the demographic structure caused by the aging of society, the prospect for stents seems to be very bright. The vascular stent market accounts for approximately 98.7% ($ 7.65 billion) of the total market (vascular and the non-vascular), and the increase of vascular disease is expected to generate continuous demand in the stent market [1].

Among vascular stents, coronary artery stents revolutionized the practice of interventional cardiology since they were first introduced in the mid-1980s [3]. The use of a stent designed to prevent acute vascular occlusion and prevent restenosis, which is a side effect of conventional balloon angioplasty, has rapidly become popular because it can prevent acute complications and improve clinical outcomes [4]. Since the initial release of these stents, there have been significant developments in their design, the most notable of which has been the introduction of drug-eluting stents (DES) [3]. Despite all the benefits of DES, concerns have been raised over their long-term safety, with reference to stent thrombosis. Newer stents have been developed in an effort to address these concerns, and examples include the following: metallic DES with durable polymers, DES with biodegradable polymers, non-polymeric DES, stents with new types of coatings, and completely biodegradable stents. Many of these stents are currently undergoing pre-clinical and clinical trials, and early results appear promising [5, 6]. First-in-man studies on small and highly selective cohorts, using multimodality intracoronary imaging, have confirmed the timing of the reabsorption process and suggested that the safety and efficacy are strong [7, 8]. Bioresorbable scaffolds (BRS) have the potential to introduce a paradigm shift in interventional cardiology, a true anatomical and functional “vascular restoration” instead of the use of artificial stiff tubes encased by a persistent metallic foreign body. Early clinical studies using the first commercially available drug-eluting bioresorbable vascular scaffold (BVS) reported very promising safety and efficacy outcomes, comparable to best-in-class second generation drug-eluting metal stents [9].

## Methods

Data for this research were gathered from primary and secondary sources as well as the databases of the Korea Institute of Science Technology Information (KISTI) located in Seoul, Korea. The key areas of the research process are described below.

### Primary research

The sources used for primary research included the databases available from the Korea Institute of Science Technology Information, past industry research services/studies, economic and demographic data, and trade and industry journals. This research was conducted to map and analyse market and technology trends.

### Secondary research

Secondary research was used to supplement and complement the primary research. Interviews were conducted with physicians and surgeons from the key hospitals and senior sale/marketing managers from stent product suppliers in South Korea.

## Results and discussion

### Concept and characteristics of stents for human blood vessels

**Fig. 1:**
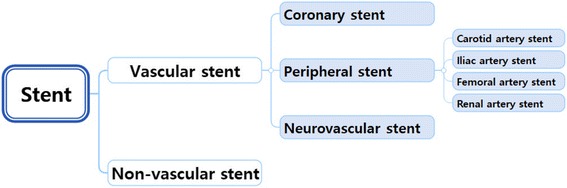
Classification of stents [17, 22]

The vascular stent is used for cardiovascular stenosis. Vascular stents are classified in specifically as coronary artery stents, peripheral stents, and neurovascular stents. Peripheral stents are used for vascular diseases other than the disease of coronary arteries. Peripheral stents include carotid artery stents, iliac artery stents, femoral artery stents, and renal artery stents [1, 2, 10] (Fig. 1).

Coronary artery stents are divided into four groups depending on the material, drug release and bioabsorbability of the product: bare metal stents (BMS), DES, covered stents, and bioresorbable stents (BRS; non-drug eluting and drug eluting BRS) [11]. Furthermore, the stents are classified into BMS (1st and 2nd generation), DES (3rd generation), and BRS (4th generation), depending on their materials and characteristics [2].

A BMS is a stent made of a metal such as stainless steel and cobalt chromium (CoCr) in the form of a slotted tube or a coil. This type of stent is used to achieve high strength and excellent stretchability. The metals that compose metal stents have poor biocompatibility with the human body and cause blood clots to be easily formed. Due to these problems, many side effects such as acute vascular occlusion and chronic complications such as restenosis are caused by endarterectomy and thrombus formation after the procedure. The use of DES has been attracting attention as a means to minimize the above side effects: DES are coated with a drug-containing polymer over the surface of metal stents [10]. The structure of the DES is usually composed of a conventional bare or coated metal stent as a platform, a polymer that controls drug release by coating the drug, and a drug that is mixed with the polymer [12]. DES is different from the human tissue, and so there is a possibility that rejection will occur when the stent is inserted into the human body [10].

The drug release stent is controlled in terms of the rate at which the drug is released, depending on the type or molecular weight of the polymer and the property of the drug incorporated therein. Materials coated on the surface of the stent should have blood compatibility, tissue compatibility, non-toxicity, and stability. Biodegradable synthetic polymers such as poly(lactic-co-glycolic acid) (PLGA), poly(L-lactic acid) (PLLA) and poly(ε-caprolactone) are mainly used, since these materials satisfy such biocompatibility. Typical DES are made by coating a biodegradable polymer on a metal platform. There is a side effect, however, if a rejection reaction occurs due to the difference from the human tissue, because phosphorylcholine groups are not detectable in the coating agent used at this time [10].

Some of the current limitations of DES include the risk of the presence of metal in the body for a lifetime, the difficulty of re-operation or surgery (which can be difficult to re-perform due to the remaining metal during multiple stenting), long-term stability problems (stent thrombosis occurs due to the residual stent after several years.) and long-term use of antiplatelet agents (increased risk of bleeding) [2].

Today, drug-eluting metal stents are considered the gold standard for the interventional treatment of coronary artery disease. While they do inhibit neointimal hyperplasia, drug-eluting metal stents have many limitations such as the risk of late and very late stent thrombosis, restriction of vascular vasomotion and chronic local inflammatory reaction due to the permanent implantation of a “metallic cage,” recognized as a foreign body. BRS are a new solution, developed to overcome the limitation of the “metallic cage.” This structure provides short-term scaffolding of the vessel and then disappears, leaving nothing behind [13].

An example of a recently introduced BRS is one made of PLLA, which is the material of the suture thread. This allowed the stent to be fully absorbed into the body 1–2 years after the operation, the intrinsic function of the coronary artery vessel was restored, and the diameter of the vessel increased over time. As the result, reoperation or surgery will be easier when restenosis occurs, long-term stability is increased by reducing the risk of stent thrombosis, and the risk of bleeding is reduced due to the shortening of the duration of antiplatelet therapy [2].

### Characteristics of stents used for human blood vessels

Stents comprise a growing industry, and along with the strong prospects for implantable medical devices overall, the stent market is recognized as an especially promising industry due to the full-fledged demographic shift toward the aging of society and the development of information and communication technologies (ICT). There are positive factors such as the expansion in governmental investment and the increase in market demand. Meanwhile, there are also negative factors such as the high market share of the developed countries and the lack of institutional support, which still play a significant role in keeping the Korean market very small, and the competition with global companies remains difficult. Therefore, efforts should be made to nurture related industries while improving the market environment [1].

The medical device industry, which includes BRS, is an industry in which safety and efficacy are of the highest importance, and is subject to intensive management by regulatory authorities. In addition, it is difficult to obtain licensing within the system to enter the market, and the success or failure of the business is greatly influenced by medical policies and regulations. On the other hand, it is a high-value-added field of the health industry that is growing in importance day by day, in which advanced technology is fused [10].

The stent market is expected to shift toward a concentration on the biodegradable stent market, which is currently the third generation of DES and compensates for the shortcomings of thrombosis. In the developed countries as well as in Korea, population aging is accelerating, and stenting due to cardiovascular diseases is continuously increasing, so the market stability and growth potential of the stent is high [10].

### Technology trends in stents used for human blood vessels

Recent developments in stent materials and drug coatings, such as applying CoCr alloys to the stents, have increased the flexibility of the stents. To prevent stent restenosis, stents coated with a drug such as an immunosuppressive agent have been developed and are now being used widely, including 20,000 cases per year in Korea alone. Recently, stents that can be completely absorbed in the blood vessels have emerged, and these are expected to revolutionize the paradigm of stenting in the future [2].

Over the past 30 years, a number of coronary artery stents have been developed, with new variations on the material and design of the stent, the type of antiproliferative drug, and the polymer coating the drug, and thus the scope of options when choosing a stent for coronary intervention has been expanded. Recently, researchers have actively conducted clinical studies of biocompatible scaffolds. Several BRS have been proposed and have now reached clinical testing, but to date, the only one for which we have only a considerable amount of clinical data available is the Absorb (Abbott Vascular, Santa Clara, CA, USA) BVS [14]. The recent developments in stent technology are mainly driven by the development of DES. Drug-releasing stent technology has three core elements: material, structure, and drugs [1]. During the last 3 years, there have been active research and development of stents for artificial heart valves, stent-grafts, and stent supports (coatings, materials and so on.) [2].

The following table shows the status of major global applications related to the stent [15] (Table 1).

**Table 1:** Status of major global applications related to stents [15]

Applicant	US applications (number)	Japan applications (number)	Europe applications (number)	Total (number)	Technological focus
Abbott Cardiovascular Systems	232	11	22	265	Stent, biocompatible polyacrylate composition
Cook Medical Technologies	77	22	32	111	Stent graft, prosthesis
Boston Scientific Scimed	77	22	32	111	Drug release stent, shape memory polymer stent
Smith & Nephew	38	8	0	47	Bioabsorbable polymer, composition for tissue regeneration
Advanced Cardiovascular Systems	48	0	0	48	Drug Delivery Device Coating

Various types of stents are being developed, mainly in the United States, Germany, India, and Japan, with variations in material development, product structure, and applied drugs. Most notably, many biodegradable stents are being developed [1].

DES is a stent with reduced in-stent restenosis (IRS) compared to BMS. DES has an average restenosis rate of less than 15% and reoperation rate of less than 6%. However, there is a high incidence of stent thrombosis compared to BMS [16]. The development of drug-eluting stent materials has been underway, to make the metal or biodegradable material harmless to the human body. Materials with biodegradability and biocompatibility have been actively developed, such as the PLLA polymer, Tyrosine- derived polycarbonate, magnesium alloy, and salicylic acid. To enhance the mechanical performance characteristics required of the stent, the main cell is divided into the connection cell and the main cell shape is divided into the diamond and wave pattern, and the open and closed structure are developed according to the width of the main cell and the connection cell. Drug-releasing stents have been developed using Sirolimus, Everolimus, and Biolimus, which are immunosuppressants, and Paclitaxel, which inhibits cell proliferation as an anticancer drug. Techniques related to this method of drug injection have been developed for the drug stent. The stent is coated with a drug-releasing stent, a biodegradable material, and a drug. The stent is naturally decomposed and absorbed after a certain period of time [1]. DES, which is a concept of local drug delivery, has been developed by coating the drug on a conventional stent and gradually releasing the drug. The localization of the drug has led to the emergence of DES as the main stent and various coating techniques are being developed to maximize the local drug effects [12].

DES are classified as first- and second-generation, depending on the drug used. The first-generation DES include Taxus (Paclitaxel-eluting stent) and Cypher (Sirolimus-eluting stent). The second-generation drug-eluting stent includes Xience prime (Everolimus-eluting stent), Resolute integrity (Zotarolimus-eluting stent), and Promus premier (Everolimus-eluting stent). It has been reported that DES have more very late stent thrombosis symptoms than BMS [17].

BRS is made of a material different from the metal used in conventional stents to maintain the inherent characteristics of the vessel and to reduce the risk of late thrombosis by preventing acute occlusion due to intimal injury and exfoliation using scaffold, prevent negative remodelling by maintaining blood vessel inner diameter for a certain period of 3–6 months, and prevent intimal hyperplasia by releasing an antiproliferative drug that prevents restenosis [4].

Therefore, BRS represent a new horizon in interventional cardiology for the treatment of coronary artery disease. The technology was introduced to overcome problems involved in the use of current metallic DES such as late IRS and the permanent caging of the vessel. The concept of BRS is to provide temporary support to the vessel during healing before being degraded and resorbed by the body, promoting restoration of the vessel vasomotion [18].

The following table describes developed metallic DES with durable polymers. Polymers such as poly(ethylene-co-vinyl acetate)(PEVA), poly(n-butyl methacrylate)(PBMA), Styrene-isobutylene-b-styrene (SIBS), Phosphorylcholine, and poly(vinlylidene fluoride-co-hexafluoropropylene) P(VDF-HFP) are used in the coating of metallic DES with durable polymers. Products applying metallic DES with durable polymers include Endeavor Resolute (Medtronic), Elixir DESyne (Elixir medical), TAXUS Element (Boston scientific), and Promus Element (Boston scientific) [17] (Table 2).

**Table 2:** Developed metallic DES with durable polymers for coronary arteries [17]

Stent	Manufacturer	Drug (Dose: μg/mm^2^)	Polymer	Polymer Thickness (μm)	Stent Platform	Strut Thickness (μm)
Cypher	Cordls Corporation	Sirolimus (1.4)	PEVA+PBMA	12.6	SS	140
Cypher select	Cordls Corporation	Sirolimus	PEVA+PBMA	N/A	SS	100
Elixir Myolimus	Elixir Medical	Myolimus (40 μg)	Methacrylate	< 3	CoCr	N/A
Endeavor	Medtronic, Inc.	Zotarolimus (10)	Phosphorylcholine	5.3	CoCr	91
Excella	Elixir Medical Corporation	Novolimus (0.85)	Methacrylate	3	CoCr	81
Promus	Boston Scientific Corporation	Everolimus (1)	Fluoropolymer	7.6	CoCr	81
Taxus Express	Boston Scientific Corporation	Paclitaxel (1)	SIBS (Translute)	16	SS	132
Taxus Liberte	Boston Scientific Corporation	Paclitaxel (1)	SIBS (Translute)	16	SS	97
Taxus Petal	Boston Scientific Corporation	Paclitaxel	SIBS (Translute)	N/A	Platinum Chromium	N/A
Xience V	Abbott Vascular	Everolimus (1)	Fluoropolymer	7.6	CoCr	81
ZoMaxx	Abbott Vascular	Zotarolimus (10)	Phosphorylcholine	5	SS/Tantalum	74

*SS* Stainless steel and *CoCr* Cobalt chromium

The following table describes developed DES with biodegradable polymers. Biodegradable polymers used in the coating of DES include polyl(actic acid)(PLA), PLGA, and poly(vinyl pyrrolidone)(PVP) (Table 3).

**Table 3:** Developed DES with biodegradable polymers for coronary arteries [17]

Stent	Manufacturer	Drug (Dose)	Polymer	Polymer Thickness (μm)	Stent platform	Strut Thickness (μm)
Axxess	Devax.Inc	Biolimus A-9 (15.6 μg/mm)	PLA	N/A	SS	112
BioMatrix	Biosensors International.ltd.	Biolimus-A9 (15.6 μg/mm)	PLA	10	SS	137
Cardiomind	CardioMind, Inc.	Sirolimus (5.2 μg/mm)	PLA + PGLA	N/A	Nitinol	61
Champion	Boston Scientific Corporation	Everolimus	PLA	N/A	SS	N/A
Corio	Cordis Corporation	Pimecrolimus	N/A	N/A	CoCr	89
CoStar	Cordis Corporation	Paclitaxel	PLGA	N/A	CoCr	89
Cura	OrbusNeich	Sirolimus (1.7 μg/mm2)	PLA + PLGA	5–10	SS	100
Excel	JW Medical Systems Ltd.	Sirolimus (195–376 μg)	PLA	N/A	SS	150
Infinnium	Sahajanand Medical Technologies Pvt.Ltd.	Paclitaxel	PLL + PLGA+PVP	N/A	SS	84
Nevo	Cordis Corporation	Sirolimus (166 μg)	PLGA	N/A	CoCr	99
Nobori	Terumo Medical corporation	Biolimus A-9	PLA	N/A	SS	120–149
Stellium	DISA Vascular (Pty) Ltd.	Paclitaxel	PLGA	N/A	CoCr	N/A
Supralimus	Sahajanand Medical Technologies Pvt.Ltd.	Sirolimus (1.4 μg/mm^2^)	PLLA+PLGA+PVP	N/A	SS	80
Symbio	Cordis Corporation	Pimecrolimus + Paclitaxel	PLGA	N/A	CoCr	89
Synchronnium	Sahajanand Medical Technologies Pvt.Ltd.	Sirolimus + Heparin	N/A	5–6	SS	60
Xtent	Xtent. Inc.	Biolimus A-9	PLA	N/A	CoCr	N/A

*SS* Stainless steel and *CoCr* Cobalt chromium

The following table is an overview of the mechanical and physical properties of these biodegradable polyesters and their degradation rates [18] (Table 4).

**Table 4:** Mechanical and physical properties of biodegradable polymers [20]

Polymer	Tg (°C)	Tm (°C)	Modulus (GPa)	Strength (MPa)	Elongation at break (%)	Degradation (months)
PLA	60	180–190	2–4	65	2–6	18–30
PDLLA	55	Amorph.	1–3.5	40	1–2	3–4
PLLA	60–65	175	2–4	60–70	2–6	> 24
PDLGA (50/50)	45	Amorph.	1–4.3	45	1–4	1–2
PLGA (82/12)	50	135–145	3.3–3.5	65	2–6	12–18
PCL	− 54	55–60	0.34–0.36	23	> 4000	24–36
PLA/PCL (70/30)	20	100–125	0.02–0.04	2–4.5	> 100	12–24
WE43 (Mg alloy)	N.A	540–640	40–50	220–330	2–20	~ 12
SS 316 L	N.A	1371–1399	193	668	40	Biostable
CoCr	N.A	~ 1454	210–235	> 1000	40	Biostable

*PLA* Polylactic acid, *PDLLA* Poly-DL-lactic acid, *PLLA* Poly-L-lactic acid, *PDLGA* Poly-DL-lactide-co-glycolide, *PLGA* Poly-lactic-co glycolide, *PCL* Polycaprolactone, *PLA/PCL* Polylactic acid/polycaprolactone, *Mg* Magnesium, *SS* Stainless steel, and *CoCr* Cobalt chromium

The following table is a summary of the main current and upcoming BRS technologies. This section will review the underlying characteristics of these developments [18] (Table 5).

**Table 5:** Summarized highlights of current BRS technologies [20]

Company	Stent name	Strut backbone material	Strut thickness (μm)	Key method	Radiopacity	Drug-eluting Property
Abbott	Absorb BVS	PLLA	150 (BVS), 100 (Next gen)	Blow molding process and orientation (stretching)	2 platinum radiopaque markers	Everolimus
Amaranth	Fortitude, Aptitude	PLLA	150, 120	Dip coating to induce linear/radial orientation	N/A	Sirolimus
ART	ART18Z	PDLLA	170 (1st gen), 140–150 (2nd gen)	Annealing	N/A	None (1st gen), Sirolimus (2nd gen)
Arterius	ArterioSorb	PLLA	140–100	Die drawing to induce orientation and alignment	Radiopaque markers	Sirolimus
Elixir	DeSolve	PLLA	150 (1st gen), 120 (2nd gen)	Annealing and quenching	Metallic markers	Novolimus
REVA medical Xenogenics	Fantom Ideal BioStent	Tyrosine PC, PAE salicylic acid	125, 175	Proprietary polymer	Iodine incorporated into the polymer or N/A	Sirolimus or Sirolimus + Salicylic acid
Envision Scientific	BIOLUTE-next	Mg alloy	120	Bio-corrodible metal and polymer coating	Radiopaque markers	Sirolimus

*BVS* Bioresorbable vascular scaffold, *PLLA* Poly-L-lactic acid, *PDLLA* Poly-DL-lactic acid, *PC* Polycarbonate, *PAE* Poly(anhydride-ester), *Mg* Magnesium, and *IBS* Iron-based bioresorbable scaffold

In patients with diabetes, the use of DES was associated with a significant reduction of target lesion restenosis without an increase in adverse events, and when compared to the use of bare metal stents and the use of a polymer-free Zotarolimus and Probucol-eluting stents, the results demonstrated comparable long-term efficacy and safety as second-generation durable polymer Zotarolimus-eluting stents [19].

### The market sizes and prospects for stents used in human blood vessels in Korea and the world

The global stent market is estimated at US $ 7.98 billion in 2016 and is expected to grow at a Compound Annual Growth Rate (CAGR) of 3.8% over the next 5 years. As of 2016, the global market for vascular stents is estimated at $ 7.22 billion, with coronary artery stents accounting for 67.3% of the vascular stent market. Among the coronary artery stents, BRS is expected to reach an annual average growth rate of 8.8% by 2020, but the global adoption rate of BRS is still low. BRS technology is in its infancy and requires long-term clinical evidence of therapeutic efficacy. Because of the high cost of BRS devices, the market share of absorbable coronary artery stents in Korea is still low at 20% [11].

The Global Vascular Stent Market Forecast (2016–2020) is shown in the following table. As of 2016, the global market for coronary artery stents is estimated at $ 5371 million, the global market for BMS at $ 508 million, the global market for BRS at $ 205 million, and the market for DES at $ 4658 million. As of 2016, the global market for neurovascular stents is estimated at US $ 141 million and the global market for peripheral stents is estimated at US $ 1507 million [11] (Table 6).

**Table 6:** Global market forecast for vascular stents (2016–2020) [Unit: million USD] [11]

Classification	Subclass	2016	2017	2018	2019	2020	CAGR(%)
Coronary Artery Stents	Bare Metal Stents	508	490	472	456	445	−3.3%
	Bioresorbable Stents	205	221	240	262	287	8.8%
	Covered Stents	140	141	144	147	152	2.1%
	Drug Eluting Stents	4658	4757	4891	5067	5282	3.2%
	Sum	5371	5468	5604	5785	6014	2.9%
Neurovascular Stents	Neurovascular Stents	141	150	160	171	182	6.7%
Peripheral Vascular Stents	Fem-pop Artery Stents	498	533	577	617	659	7.3%
	Iliac Artery Stents	833	889	963	1026	1094	7.1%
	Infrapop Artery Stents	176	188	204	217	225	6.3%
	Total	1507	1611	1744	1861	1979	7.0%

Vascular stents account for approximately 98.7% of the total stent market. Coronary artery stents used for cardiovascular diseases account for about 76.1% of the stent market for blood vessels, and the market growth rate for neurovascular stents is 9.7%. However, the market size for neurovascular stents is relatively low at 1.2% compared with the cardiovascular stent, because the demand for related diseases has been low [2].

The market value of coronary artery stents is expected to decline slowly in the US and five EU countries (France, Germany, Italy, Spain, and UK) due to the decline in the average selling price and the decrease in the number of BMS and DES used in surgeries. On the other hand, the coronary stent market is expected to grow steadily in the Asia-Pacific (9.3%) and South America (3.1%), and most manufacturers are focused on the economic growth of countries in Asia, which is regarded as the main target market. China, which has a high proportion of elderly people in its population who account for a large number of procedures, is expected to become a major market for coronary stents. Although many stent manufacturers are devoted to the research and development of BRS, which represents a fast-growing market segment, BRS has a low adoption rate for clinical care because BRS technology requires long-term clinical evidence of therapeutic effects at an early stage. The following table presents data regarding market sizes and prediction of coronary artery stent usage by continent (2016–2023) [11] (Table 7).

**Table 7:** Current sizes and forecasts of the market for coronary artery stents by continent (2016–2023) [Unit: million USD [11]

Continent	2016	2017	2018	2019	2020	2021	2022	2023	CAGR (%)
Asia-Pacific	2715	2926	3169	3448	3771	4143	4574	5072	9.3%
Europe	917	888	861	836	810	786	765	740	−3.0%
Middle East and Africa	65	64	63	63	62	61	60	58	−1.7%
South and Central America	115	118	122	126	130	134	138	142	3.1%
North America	1559	1471	1389	1312	1241	1175	1114	1057	−5.4%
Global	5371	5468	5604	5785	6014	6300	6651	7068	4.0%

In the global market for coronary artery stents, the top three companies, Abbott, Boston Scientific and Medtronic, account for 76% of the market share. In the coronary artery stent market, which constitutes the largest portion of the stent market, Abbott Vascular Inc. leads the market with approximately 50% of market share, followed by Boston Scientific and Medtronic, Inc. These companies also have large market shares. In the peripheral stent market, companies such as Cordis Corporation, Abbott, and Boston Scientific have high market shares, and Abbott Vascular Inc. and Boston Scientific are likely to lead the market for cerebrovascular stents [1, 10].

Globally, as of October 2017, the development of the coronary artery stent pipeline was in the clinical stage, with 48 clinical cases accounting for 50% of the total pipeline. Currently, most of the coronary artery stent pipeline products are DES (46%), mainly in the clinical phase (60%). The following table shows global stent pipelines for coronary arteries under development [11] (Table 8).

**Table 8:** Global stent pipelines for coronary arteries under development [11]

Classification	Bare Metal Stents	Drug Eluting Stents	Bioresorbable Stents	Coronary Artery Stents
Early development	0	6	6	7
Pre-Clinical	0	7	5	1
Clinical	9	25	11	3
In Approval Process	2	4	3	2
Sum	11	42	25	13

As competition intensifies, there has been much litigation regarding the infringement of intellectual property rights. One example, according to a patent litigation study by PWC in May 2017, was the infringement lawsuit filed by Cordis against Medtronic Vascular in 2005, in which $ 595 million of damages were awarded, and another was a patent infringement case filed by physician Bruce N. Saffran against Johnson & Johnson, in which $ 482 million of damages were awarded [20].

The following table shows the size and prospects of the Korean market for vessel stents [1] (Table 9).

**Table 9:** Size and forecast of the Korean market for vessel stents (2015–2020) [Unit: million Korean won] [1]

2015	2016	2017	2018	2019	2020	CAGR (%)
116,237	115,282	114,335	113,397	112,465	111,542	−0.8

In the Korea, stents for blood vessels account for most of the market. In Korea, the market size for stents used in blood vessels was estimated to be 145 billion won as of 2016. Over 92% of the total Korean market for stents is comprised of imports. By 2016, Korea’s stent imports amounted to 122 billion won, accounting for 4.1% of the total import of medical devices (299.4 billion won) in Korea. In Korea, practice of coronary artery stent implantation has steadily increased to 58,064 cases as of 2013, with an average annual increase of 5.3%. [1, 17, 11, 21].

The Korean stent market is dominated by the top three importers, namely Medtronic Korea, Korea Abbott and Boston Scientific Korea, which account for about 70% of the total market. Meanwhile, the top-ranked domestic Korean company has a market share of about 5%. The market is thus highly dependent on foreign vendors [1]. As of 2016, there were 16 vessel stent importers in Korea, including Medtronic, Boston Scientific, Abbott Vascular, BIOTRONIK AG, Ashitaka Factory of Terumo Corporation, Angiomed GmbH & Co, and Medizintechnik KG. Korean manufacturers of blood vessel stents include Osstem Cardiotec and GENOSS [2, 11]. In addition, SUNTECH has acquired the cardiovascular stent business of KOSWIRE and Daewoong Pharma is also pursuing the cardiovascular stent business through its subsidiary CSII BIO. Therefore, competition in the market is expected to intensify [1].

The following table lists stent related companies in Korea and overseas and their respective core technologies [1] (Table 10).

**Table 10:** Stent related companies in Korea and overseas and their core technologies [1]

Company	Main Technology
Johnson & Johnson (MD&D)	Orthopedic (spine, etc.) implants, minimally invasive surgical instruments, wound sutures, contact lenses, blood glucose meters, catheters, stents, etc.
Medtronic, Inc.	Defibrillator, pacemaker, stent, catheter, spine regeneration, bone graft material, etc.
Covidien plc (MD)	Laparoscopic, surgical stapler, electrosurgical instruments, stents and orthopedic supplies
Boston Scientific Corporation	Access devices, balloon dilators, catheters, stents, slings
Abbott Vascular Inc	Vessel closure, structural heart, humanitarian use devices, peripheral intervention
Biotronik	Cardiac rhythm management, peripheral vascular intervention
REVA	Medical unique bioresorbable polymers
Zorion Medical	ZMED devices (biodegradable stent)
S3V Vascular Technologies	Cardio vascular, critical care
Japan Stent Technology	Biodegradable stents
OrbusNeich	Dual therapy stents, bio-engineered stents, bare metal stents, semi-compliant balloons, non-compliant balloons, specialty balloons
Taewoong Medical	Non-vessel stents (gastroenterology, urology, pulmonary, general surgery, cardiology)
MITECH CORPORATION	Stents, GI accessories
Sewoon Medical	Non-vessel stents

## Conclusions

There can be variances in commercial prospects within the industry depending on the market and scope of stent products, but most global market analysis reports forecast the stent market to grow at a somewhat rapid rate. Fairly rapid growth is anticipated especially in the BRS market. In comparison to the sales growth rate for medical devices in general, the stent technology market is judged to have a higher growth potential.

It is expected that social costs will increase as the population ages rapidly. Therefore, it is necessary to nurture the medical device industry to better treat the diseases of elderly patients and maintain a robust medical device industry. However, physicians who deal with implantable medical devices have a strong social awareness of the preference for foreign products. There are factors that make it challenging to enter the market through new technology development, such as aspects of governmental policy and institutions, safety and efficacy evaluation through non-clinical animal testing, clinical trial approval procedures, and the difficulty of clinical trials.

In the global market for coronary artery stents, the top three companies, Abbott, Boston Scientific, and Medtronic, have a 76% share and enjoy a monopoly position in the market, making the entry of new companies difficult. Korea also has a chronic trade deficit in the stent field. Although many stent manufacturers are devoted to the research and development of BRS, the fast-growing market segment, the rates of the adoption of BRS in clinical practice are low, as BRS technology requires long-term clinical evidence of therapeutic effects in its early stages.

Vascular stents account for approximately 98.7% of the total stent market. Coronary artery stents used for cardiovascular diseases account for about 76.1% of the stent market for blood vessels, and the market growth rate for neurovascular stents is 9.7%. However, the market size for neurovascular stents was relatively low at 1.2% compared with cardiovascular stents, because the demand arising from related diseases was low.

The coronary artery stent market value is expected to decline slowly in the US and 5 EU countries (France, Germany, Italy, Spain, and UK) due to the decrease in the average selling price and the number of BMS and DES used in surgeries. On the other hand, the coronary stent market is expected to grow steadily in the Asia-Pacific (9.3%) and South America (3.1%), and most manufacturers are focused on the economic growth of countries in Asia, which is regarded as the main target market. China has a high proportion of elderly in its population who account for the demand for procedures and is expected to become a major market for coronary stents.

The prospect for stents seems to be very bright due to current shift toward an aging demographic structure.
